# Controlling microbial contamination during hydrolysis of AFEX-pretreated corn stover and switchgrass: effects on hydrolysate composition, microbial response and fermentation

**DOI:** 10.1186/s13068-015-0356-2

**Published:** 2015-11-14

**Authors:** Jose Serate, Dan Xie, Edward Pohlmann, Charles Donald, Mahboubeh Shabani, Li Hinchman, Alan Higbee, Mick Mcgee, Alex La Reau, Grace E. Klinger, Sheena Li, Chad L. Myers, Charles Boone, Donna M. Bates, Dave Cavalier, Dustin Eilert, Lawrence G. Oates, Gregg Sanford, Trey K. Sato, Bruce Dale, Robert Landick, Jeff Piotrowski, Rebecca Garlock Ong, Yaoping Zhang

**Affiliations:** DOE Great Lakes Bioenergy Research Center, University of Wisconsin-Madison, Madison, WI USA; DOE Great Lakes Bioenergy Research Center, Michigan State University, East Lansing, MI USA; RIKEN Center for Sustainable Resource Science, Wako, Saitama Japan; Department of Computer Science and Engineering, University of Minnesota-Twin Cities, Minneapolis, MN USA; Terrence Donnelly Centre for Cellular and Biomolecular Research, University of Toronto, Toronto, ON Canada

**Keywords:** Biomass feedstock, Lignocellulosic hydrolysate, Fermentation, Chemical genomics, Inhibitors, Sterility, *Saccharomyces cerevisiae*, *Zymomonas mobilis*

## Abstract

**Background:**

Microbial conversion of lignocellulosic feedstocks into biofuels remains an attractive means to produce sustainable energy. It is essential to produce lignocellulosic hydrolysates in a consistent manner in order to study microbial performance in different feedstock hydrolysates. Because of the potential to introduce microbial contamination from the untreated biomass or at various points during the process, it can be difficult to control sterility during hydrolysate production. In this study, we compared hydrolysates produced from AFEX-pretreated corn stover and switchgrass using two different methods to control contamination: either by autoclaving the pretreated feedstocks prior to enzymatic hydrolysis, or by introducing antibiotics during the hydrolysis of non-autoclaved feedstocks. We then performed extensive chemical analysis, chemical genomics, and comparative fermentations to evaluate any differences between these two different methods used for producing corn stover and switchgrass hydrolysates.

**Results:**

Autoclaving the pretreated feedstocks could eliminate the contamination for a variety of feedstocks, whereas the antibiotic gentamicin was unable to control contamination consistently during hydrolysis. Compared to the addition of gentamicin, autoclaving of biomass before hydrolysis had a minimal effect on mineral concentrations, and showed no significant effect on the two major sugars (glucose and xylose) found in these hydrolysates. However, autoclaving elevated the concentration of some furanic and phenolic compounds. Chemical genomics analyses using *Saccharomyces cerevisiae* strains indicated a high correlation between the AFEX-pretreated hydrolysates produced using these two methods within the same feedstock, indicating minimal differences between the autoclaving and antibiotic methods. Comparative fermentations with *S. cerevisiae* and *Zymomonas mobilis* also showed that autoclaving the AFEX-pretreated feedstocks had no significant effects on microbial performance in these hydrolysates.

**Conclusions:**

Our results showed that autoclaving the pretreated feedstocks offered advantages over the addition of antibiotics for hydrolysate production. The autoclaving method produced a more consistent quality of hydrolysate, and also showed negligible effects on microbial performance. Although the levels of some of the lignocellulose degradation inhibitors were elevated by autoclaving the feedstocks prior to enzymatic hydrolysis, no significant effects on cell growth, sugar utilization, or ethanol production were seen during bacterial or yeast fermentations in hydrolysates produced using the two different methods.

**Electronic supplementary material:**

The online version of this article (doi:10.1186/s13068-015-0356-2) contains supplementary material, which is available to authorized users.

## Background

Biofuel production from lignocellulosic biomass provides a sustainable route to energy security. The common biological route for biofuel production consists of three major steps: biomass pretreatment, enzymatic hydrolysis and microbial fermentation. A chemical, mechanical, or combined pretreatment is necessary to increase enzyme access to the cellulose and hemicellulose fractions during enzymatic hydrolysis. Although there are many pretreatment options available, each has its own limitations and some methods only work well with certain kinds of feedstocks [1–5]. Furthermore, all pretreatments inevitably generate degradation inhibitors [6–8], which can hinder microbial performance during fermentation [9–13]. In some cases, the reagents or organic solvents used for pretreatment are also toxic to microorganisms [14, 15]. One pretreatment, Ammonia Fiber Expansion (AFEX), is a high-pressure alkaline pretreatment [16, 17] that is effective on corn stover (CS) [18–20], switchgrass (SG) [21–23], and a variety of other feedstocks [16, 24–27]. AFEX is able to preserve most nutrients naturally found in the plant biomass [19, 28] and also generates fewer degradation products compared to dilute acid pretreatment, including total carboxylic acids, furanic aldehydes, and phenolics [8].

During enzymatic hydrolysis, bacterial contamination (predominantly *Lactobacillus* sp.) can be a major problem, leading to significant reductions in sugar and ethanol yields, consumption of nutrients, and generation of lactic and acetic acids, which can inhibit the fermentative organisms [29, 30]. Although efforts can be made to sterilize equipment and process inputs, preventative measures may not be sufficient to control contamination. This is particularly true if the contamination arrives with the feedstock and manages to survive the pretreatment process [29]. Ammonia is an effective disinfectant and has been used to sterilize feedstocks prior to ethanol production [31]. In a previous study, no contamination was observed in AFEX-pretreated biomass immediately following pretreatment, with significant preventative measures taken during post-processing to prevent contamination [32]. However, even with these precautions there is often the accumulation of lactate during hydrolysate production without some additional control [31]. Elimination of contamination in our case is particularly crucial as the hydrolysate being produced is used for comparative microbial research and needs to have consistent characteristics and quality. A variety of methods can be used to control microbial contamination during hydrolysis and fermentation including (1) tailoring the levels of pretreatment degradation products to control contamination while limiting negative impacts on the fermentative organism [33]; (2) autoclaving the pretreated biomass prior to enzymatic hydrolysis [12, 32]; (3) adding antibiotics to the hydrolysis and/or fermentation [29, 30]; (4) pasteurizing the fermenters and their contents mid-way through the process [29]; (5) spiking the hydrolysis/fermentation with high concentrations of ethanol to inhibit growth of contaminating organisms [29, 34, 35]; and (6) expressing bacteriophage lytic enzymes (endolysins) in the fermentative organism [36]. Of these methods, only autoclaving the pretreated biomass and adding antibiotics are suitable for enzymatic hydrolysis, and have been demonstrated to consistently control contamination [12, 29, 32, 37]. Unfortunately, autoclaving the biomass can potentially generate unique inhibitors or alter the concentration of known inhibitors, which could negatively impact fermentation. And although antibiotics can be very effective, there are some strong negatives associated with their widespread use: they are costly and carry serious environmental risks including the development of antibiotic-resistant strains and transfer of this resistance to other organisms [30].

To determine the most effective and consistent method for control of contamination during hydrolysate production, we compared hydrolysates made from autoclaved AFEX-pretreated feedstocks to those made from non-autoclaved pretreated feedstocks, for which microbial growth was controlled by the addition of antibiotics during the enzymatic hydrolysis. To ensure that the method was consistently able to control contamination regardless of the feedstock, hydrolysates were generated from both corn stover and switchgrass with both methods. To determine whether there were any practical differences in the hydrolysate quality and fermentation performance between the autoclaving and antibiotics methods, hydrolysates were evaluated in terms of their chemical composition, and compared by chemical genomics and microbial fermentation using the ethanologens *Saccharomyces cerevisiae* and *Zymomonas mobilis*.

## Results and discussion

### Effectiveness of antibiotic gentamicin to control contamination in ACSH and ASGH production

**Fig. 1:**
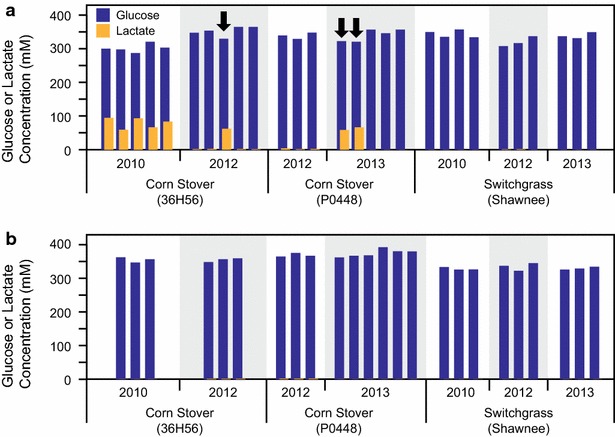
Concentrations of lactate and glucose in different batches of ACSH and ASGH produced from different years of corn stover and switchgrass: **a** by the NAC method in the presence of gentamicin and **b** by the AC method. Each *bar* represents an individual batch of hydrolysate. An *arrow* indicates a higher lactate and lower glucose concentration in the hydrolysate compared with other batches of hydrolysate produced from the same feedstock. Because of lactate production, all batches of hydrolysate produced from 2010 CS showed a lower level of glucose than other ACSH produced using the NAC method

Using the non-autoclaved (NAC) method with gentamycin, we generated several batches of ACSH and ASGH from four different corn stover feedstocks (Pioneer 36H56 cultivar from year 2010 and 2012, and Pioneer P0448 cultivars from 2012 and 2013) and three switchgrass feedstocks (Shawnee variety, harvested from years 2010, 2012, and 2013). Although gentamicin could effectively control the contamination in switchgrass hydrolysate production, this was not true for corn stover, especially the material harvested in 2010 (Fig. 1a). High levels of lactate were detected in all five batches of hydrolysates produced from 2010 CS, as well as in one batch of hydrolysate from 2012 CS and two batches of hydrolysates from 2013 CS. As expected, the high lactate production correlated to lower glucose concentrations in these hydrolysates (2012 CS 36H56: *R* = −0.86, *p* = 0.062; 2013 CS P0448: *R* = −0.97, *p* = 0.006) (Fig. 1). In contrast to the NAC method, the autoclaved (AC) method was able to control contamination, with extremely low levels of lactate for hydrolysates produced from both 2012 CS varieties (<2 mM) and no detectable lactate in all other hydrolysates (Fig. 1b). To further compare the differences between the two methods, we evaluated 12 batches of hydrolysates produced from 2012 CS and 2010 SG (three batches of NAC hydrolysate and three batches of AC hydrolysate for each feedstock), with fermentations conducted in biological triplicate.

### Comparison of the concentration of major sugars and acids in ACSH and ASGH

The concentrations of glucose, xylose and some organic acids and alcohols in the 12 batches of ACSH and ASGH were analyzed by HPLC (Table 1). There were no significant differences (*p* < 0.05) in glucose, xylose, organic acid, or alcohol concentrations within a given feedstock, regardless of the method used for control of microbial growth, either by the AC or NAC method. However, the glucose concentration was slightly lower in ASGH compared to that in ACSH, even when steps were taken to improve hydrolysis (increasing AFEX ammonia loading and hydrolysis enzyme and solids loadings). Unlike acetic acid, which is produced upon hydrolysis from plant polysaccharides, lactic acid is less common in plant materials, being produced through fermentative pathways under anoxic conditions [38], and its presence in the hydrolysate generally indicates microbial contamination at some stage of the process. As shown in Table 1, a small amount of lactate was found in ACSH, but not in ASGH. Because lactate was also detected in ACSH produced by the AC method, it is likely that this lactate was produced in the corn stover prior to hydrolysis. There was no difference in the concentration of lactate within the same feedstock hydrolysate using either the AC or NAC methods, indicating effective control of microbial contamination by the antibiotic gentamicin in the hydrolysate production using these two feedstocks.

**Table 1:** Composition of AFEX-treated corn stover hydrolysate (ACSH) and AFEX-treated switchgrass hydrolysate (ASGH) produced by either AC or NAC method

Components	AC-ACSH	NAC-ACSH	AC-ASGH	NAC-ASGH
Major carbohydrates and acids (mM)				
d-Glucose	355.0^a^ ± 5.8	357.4^a^ ± 8.6	328.7^b^ ± 4.2	343.9^ab^ ± 11.3
d-Xylose	211.2 ± 3.6	209.1 ± 5.6	208.0 ± 2.5	223.4 ± 10.6
Succinate	0.6 ± 0.1	0.7 ± 0.2	0.4 ± 0.1	0.6 ± 0.1
Lactate	1.4^a^ ± 0.1	1.6^a^ ± 0.4	0^b^	0^b^
Glycerol	5.4^a^ ± 0.7	5.4^a^ ± 0.5	3.6^b^ ± 0.4	4.0^b^ ± 0.7
Formate	2.8^bc^ ± 2.4	1.6^c^ ± 1.1	7.6^a^ ± 2.0	5.7^ab^ ± 1.5
Acetate	31.4^b^ ± 2.7	30.7^b^ ± 2.0	42.1^a^ ± 1.6	40.9^a^ ± 1.8
Acetamide	107.5 ± 13.0	107.0 ± 6.8	88.9 ± 2.5	111.6 ± 17.1
Abundant minerals and anions (mM)				
P	17.4^a^ ± 1.0	16.6^a^ ± 0.4	12.5^b^ ± 0.9	12.0^b^ ± 0.4
K	39.6^b^ ± 1.0	39.1^b^ ± 2.0	52.3^a^ ± 1.6	53.2^a^ ± 2.1
Ca	4.5^a^ ± 0.2	4.6^a^ ± 0.2	0.6^b^ ± 0.0	0.4^b^ ± 0.1
Mg	11.7^b^ ± 0.3	11.6^b^ ± 0.4	14.8^a^ ± 0.8	15.1^a^ ± 0.8
S	4.3^ab^ ± 0.2	4.1^b^ ± 0.4	4.8^a^ ± 0.1	4.7^a^ ± 0.1
Na	1.7^b^ ± 0.0	1.6^b^ ± 0.1	3.0^a^ ± 0.1	3.0^a^ ± 0.1
Cl	59.3^b^ ± 3.6	57.3^b^ ± 1.0	80.6^a^ ± 1.1	76.4^a^ ± 8.7
PO_4_	17.4^a^ ± 1.0	16.6^a^ ± 0.4	12.5^b^ ± 0.9	12.0^b^ ± 0.4
SO_4_	4.3^ab^ ± 0.2	4.1^b^ ± 0.4	4.8^a^ ± 0.1	4.8^a^ ± 0.1
NH_4_-N	49.7 ± 3.8	52.5 ± 5.5	51.2 ± 9.0	54.8 ± 7.1
Total N	252.7^a^ ± 6.8	252.5^a^ ± 3.6	226.4^b^ ± 8.7	219.9^b^ ± 10.6
Trace minerals and anions (μM)				
Zn	15.6^a^ ± 0.6	18.4^a^ ± 2.0	7.1^b^ ± 0.2	8.2^b^ ± 0.8
B	19.1^a^ ± 2.8	5.5^Ab^ ± 3.4	6.8^b^ ± 1.1	<3.7^Ab^
Mn	88.8^a^ ± 14.1	85.4^a^ ± 14.8	38.9^b^ ± 3.5	42.8^b^ ± 2.7
Fe	23.8^ab^ ± 3.5	31.3^a^ ± 2.5	16.9^b^ ± 2.5	17.4^b^ ± 3.9
Cu	2.5^a^ ± 0.7	2.2^a^ ± 0.3	0.9^b^ ± 0.1	1.1^b^ ± 0.2
Al	9.9 ± 3.6	9.8^A^ ± 7.2	8.8^A^ ± 5.4	15.1 ± 2.3
Cd	<0.1^A^	<0.1^A^	<0.1^A^	<0.1^A^
Co	<0.1^A^	<0.1^A^	<0.1^A^	<0.1^A^
Cr	4.8 ± 0.7	10.5 ± 3.5	5.4 ± 1.2	8.8 ± 3.9
Mo	0.3^a^ ± 0.1	0.1^Ab^ ± 0.0	0.4^a^ ± 0.1	0.1^Ab^ ± 0.0
Ni	2.6^ab^ ± 0.3	2.9^a^ ± 0.5	1.4^b^ ± 0.4	1.4^b^ ± 0.7
Pb	<0.2^A^	<0.2^A^	<0.2^A^	<0.2^A^
Li	0.4^A^ ± 0.3	0.4 ± 0.1	0.4^A^ ± 0.3	0.4^A^ ± 0.3
Br	<0.1^A^	<0.1^A^	<0.1^A^	<0.1^A^
NO_2_	<0.2^A^	<0.2^A^	<0.2^A^	<0.2^A^
Lignotoxins and other inhibitors (μM)				
Furfural	67.2^a^ ± 19.7	36.7^b^ ± 11.5	54.3^ab^ ± 2.3	32.7^b^ ± 4.1
Benzamide	3.1^b^ ± 0.3	2.6^b^ ± 0.2	6.6^a^ ± 0.8	5.9^a^ ± 0.3
Coumaroyl amide	3152.1^a^ ± 366.0	1937.6^b^ ± 97.3	1873.0^b^ ± 63.3	982.5^c^ ± 65.5
Feruloyl amide	1529.0^a^ ± 162.6	1043.7^b^ ± 31.8	667.3^c^ ± 9.4	375.2^d^ ± 24.6
4-Hydroxybenzamide	12.0^a^ ± 1.3	12.3^a^ ± 0.6	10.3^a^ ± 1.1	7.1^b^ ± 0.5
Syringamide	42.5^a^ ± 11.1	24.8^b^ ± 2.4	28.7^b^ ± 3.7	17.6^b^ ± 1.7
Vanillamide	125.6^a^ ± 12.6	61.6^bc^ ± 6.5	76.8^b^ ± 8.7	51.8^c^ ± 6.4
Acetovanillone	40.0^a^ ± 5.3	33.9^ab^ ± 4.6	28.8^b^ ± 0.6	18.2^c^ ± 0.5
Acetosyringone	0.2^Ab^ ± 0.1	0.5^Ab^ ± 0.4	1.8^a^ ± 1.0	1.2^ab^ ± 0.2
5-Hydroxymethylfurfural (HMF)	1.1^a^ ± 0.3	0.6^b^ ± 0.0	1.1^a^ ± 0.2	0.4^b^ ± 0.0
4-Hydroxybenzaldehyde	21.6^a^ ± 3.5	13.7^b^ ± 0.8	19.6^a^ ± 3.4	10.1^b^ ± 0.7
4-Hydroxyacetophenone	1.9^ab^ ± 0.6	1.0^c^ ± 0.2	2.3^a^ ± 0.5	1.2^bc^ ± 0.1
Syringaldehyde	7.4^a^ ± 1.7	1.8^b^ ± 0.2	5.4^a^ ± 1.7	0.9^b^ ± 0.1
Vanillin	141.5^a^ ± 14.5	73.8^b^ ± 5.0	76.6^b^ ± 17.8	27.5^c^ ± 1.3
4-Hydroxybenzyl alcohol	<0.2^Ab^	0.3^Ab^ ± 0.3	0.5^Ab^ ± 0.6	1.4^a^ ± 0.1
Vanillyl alcohol	0.3^b^ ± 0.0	0.6^ab^ ± 0.3	0.5^ab^ ± 0.1	0.8^a^ ± 0.1
Azelaic acid	21.4 ± 0.9	22.1 ± 1.3	19.5 ± 1.4	21.9 ± 1.5
Benzoic acid	168.3^b^ ± 39.7	179.3^b^ ± 19.3	291.7^a^ ± 22.5	286.6^a^ ± 12.6
Coumaric acid	489.1^a^ ± 142.5	484.6^a^ ± 80.4	268.2^ab^ ± 70.2	190.3^b^ ± 6.2
3,4-Dihydroxybenzoic acid	4.5^bc^ ± 1.2	2.6^c^ ± 0.6	11.1^a^ ± 2.3	6.7^b^ ± 1.3
Ferulic acid	25.2^a^ ± 4.8	26.6^a^ ± 3.5	19.7^a^ ± 4.5	9.5^b^ ± 2.2
3-Hydroxybenzoic acid	0.2^Ab^ ± 0.1	0.2^Ab^ ± 0.1	0.7^a^ ± 0.3	0.5^ab^ ± 0.1
4-Hydroxybenzoic acid	48.1^a^ ± 10.1	37.4^ab^ ± 3.7	35.6^ab^ ± 6.8	30.2^b^ ± 1.9
Sinapic acid	1.2^b^ ± 0.2	1.6^a^ ± 0.2	0.2^Ac^ ± 0.0	0.1^c^ ± 0.0
Syringic acid	9.6^a^ ± 1.4	6.9^b^ ± 1.0	10.1^a^ ± 0.5	6.2^b^ ± 0.5
Vanillic acid	29.0^c^ ± 4.2	22.8^c^ ± 2.9	55.2^a^ ± 3.7	39.8^b^ ± 3.9
8,8′ Diferulic acid	3.8^a^ ± 1.0	2.1^b^ ± 0.5	2.1^b^ ± 0.8	0.9^b^ ± 0.2
8,5′ Diferulic acid	0.3^a^ ± 0.1	0.1^Ab^ ± 0.0	0.2^b^ ± 0.0	<0.2 ^Ab^
8,8′ Diferulic acid (THF)	0.1^b^ ± 0.0	0.1^a^ ± 0.0	0.1^Ab^ ± 0.0	0.1^b^ ± 0.0
8-O-4′ Diferulic acid	1.4^a^ ± 0.2	1.2^a^ ± 0.4	0.9^ab^ ± 0.2	0.5^b^ ± 0.1

The data are reported as average ± standard deviation of at least three biological replicates. Values in each row that have different lowercase letter superscripts are statistically different based on Tukey’s 95 % confidence intervals. Rows with no superscript have no statistical difference between the values

^A^One or more replicates were below the limit of detection (LOD). Where all replicates were below the limit, the LOD is reported with no standard deviation. When fewer than all were below the limit, the values were recalculated as LOD/√2. These recalculated values were used to determine the mean, standard deviation, and statistical differences

As shown in Table 1, significantly higher (*p* < 0.05) concentrations of formate and acetate were found in ASGH compared to ACSH. The acetamide concentration was roughly 2–3.5× higher than the acetate concentration in both ACSH and ASGH, but with no significant difference in concentration between the two feedstocks or different microbial control methods. In a previous study on AFEX-treated corn stover, an estimated 8–9 % of total acetyl linkages on hemicellulose were converted to acetic acid, with the remainder converted to acetamide [8]. Acetamide has been shown to be less inhibitory than acetic acid on growth and xylose utilization of both *S. cerevisiae* and *Escherichia coli* [39, 40].

### Comparison of the mineral concentrations in ACSH and ASGH

Autoclaving corn stover and switchgrass prior to hydrolysis showed no significant effect on the concentration of minerals compared to the NAC method, except in the case of molybdenum, for which the autoclaved concentration was statistically higher (~0.30 vs 0.08 μM; *p* < 0.05) (Table 1). However, more differences were seen between ACSH and ASGH. Compared to ASGH, ACSH showed an approximately eightfold higher concentration of Ca, an approximate twofold higher concentration of Zn, Mn, Cu, Fe, and Ni, plus a slightly higher concentration of P. In contrast, ASGH had statistically higher concentrations of K, Mg, Na, and Cl compared to ACSH (*p* < 0.05). The chloride concentrations of both hydrolysates were the highest of the compounds measured, with the exception of total nitrogen. However, much of this is contributed by HCl added to adjust the pH of the hydrolysate prior to the addition of enzymes, as described in the “Methods” section.

### Comparison of lignocellulose-derived inhibitors

To determine whether autoclaving AFEX-pretreated feedstocks increases the concentration of phenolic and furanic inhibitors, more than 30 of these compounds, including aromatic acids, amides, and aldehydes were quantified. As shown in Table 1, autoclaving AFEX-pretreated corn stover and switchgrass significantly increases the concentration of furfural and several lignotoxins (*p* < 0.05), including 5-hydroxymethylfurfural (HMF), syringic acid, 4-hydroxyacetophenone, coumaroyl amide, feruloyl amide, vanillamide, vanillin, 4-hydroxybenzaldehyde, and syringaldehyde. Autoclaving would likely cleave some of the residues within the lignin polymer, and degrade some of the soluble and polymeric sugars to furanic aldehydes (furfural and HMF). However, the neutral to alkaline pH during autoclaving (pH 7 or higher) limits the degradation compared to that would occur under more acidic conditions. It was recently reported that ACSH contains a high concentration of HMF (1.1 mM, 1000× higher than our results) [40]. The different level of HMF could be explained several ways, including that the corn stover was from a different variety with a different starting composition, and that the pretreatments were conducted at different temperatures. However, it may also have resulted from different quantitation methods. In the previous paper HMF concentration was calculated based on the amount of HMF in the dry pretreated biomass [40], rather than in hydrolysates that had been measured directly, as in this study.

Comparison of the two different feedstocks showed that ACSH contains statistically higher levels of many lignotoxins (*p* < 0.05), especially coumaric acid, coumaroyl amide, feruloyl amide, acetovanillone, sinapic acid, vanillamide, and vanillin than ASGH does (Table 1). However, ASGH showed higher levels of 3,4-dihydroxybenzoic acid, benzoic acid, benzamide, and vanillic acid than ACSH.

A number of inhibitors (acids, aldehydes, and inorganic ions) have been evaluated for their inhibitory effect on microorganisms during fermentation. Some of those compounds present in our hydrolysate have been shown to have a negative impact on *Z. mobilis* and *S. cerevisiae* growth; however, concentrations in the mM range were required for inhibition, which is an order of magnitude or higher than that is found in our hydrolysates [10, 13].

### Biological fingerprinting of hydrolysates using chemical genomics

As described previously, we have used a collection of *S. cerevisiae* deletion mutants to study different hydrolysates and their inhibitors following a chemical genomics (CG) approach [41]. This process offers a means of comparing the biological response to different hydrolysates via the differential fitness of yeast mutants in each hydrolysate compared to control media. The degree of correlation of chemical genomic profiles between hydrolysates is a measure of hydrolysate similarity, and the response of individual mutants gives insight into hydrolysate-specific inhibitors and nutrient limitations. Here we used this approach to compare the difference in the fitness response of yeast mutants, in ACSH and ASGH produced by AC and NAC methods.

**Fig. 2:**
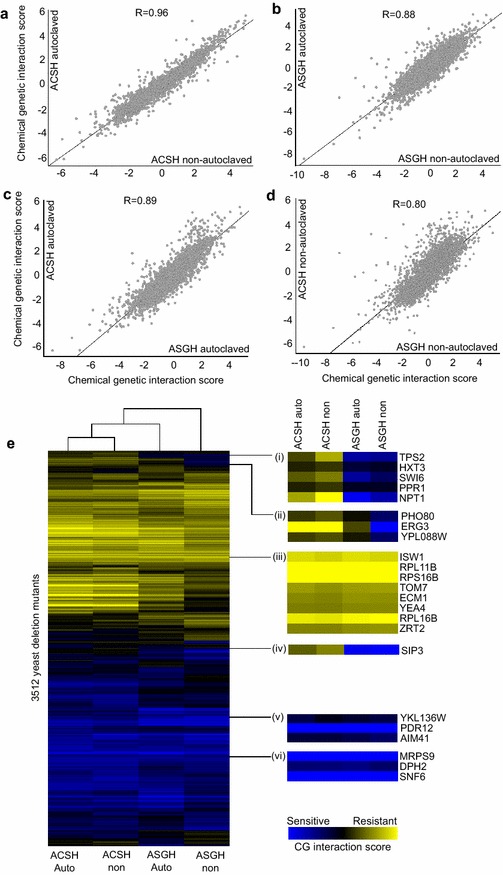
Biological fingerprinting via chemical genomics to assess hydrolysate variation. We grew the genome-wide yeast deletion mutant collection in the four different hydrolysate batches, or a synthetic hydrolysate (SynH) control (*n* = 3). The abundance of each mutant is assessed by sequencing of the strain specific barcodes, and this compared to the abundance in the SynH control allows us to determine sensitivity (*blue*) or resistance (*yellow*) of specific mutants to the hydrolysate conditions (chemical genetic interaction score). The performance of all mutants in a particular condition is the chemical genomic profile. Hydrolysates produced from corn stover or switchgrass via AC or NAC had highly correlated chemical genomic profiles, indicating little variation in the biological response between methods (**a**–**d**). Chemical genomic profiles were clustered as a heat map of chemical genomics interaction score in four different hydrolysates (**e**), and both biomass types had greater correlation with each other irrespective of the production methods. When we zoom in on particular gene clusters (*i*–*vi*), we see certain gene mutants were commonly responsive across all hydrolysates (**e** *iii*, *v*, *vi*), and others demonstrated a feedstock-specific response (**e**, *i*, *ii*, *iv*), showing sensitivity to switchgrass, but resistance in the ACSH (e.g., *ERG3* in *ii*)

As shown in Fig. 2, panel a–d, where each point represents the CG interaction score of a single deletion mutant, we found that batches of ACSH produced by the different methods were highly correlated (*R* = 0.96) (Fig. 2a), indicating that autoclaving had little impact on the biological response of yeast, despite elevated concentrations of some of the lignotoxins. A slightly lower correlation (*R* = 0.88) was found in ASGH produced by the two different methods (Fig. 2b), which is probably due to the lower correlation between three replicate samples of NAC-produced ASGH. As shown in Additional file 1: Figure S1, three biological replicates of ACSH batch 763, 764 and 778 showed high correlation (*R* = 0.88–0.91), but three biological replicates of ASGH batch 769, 770 and 781 showed lower correlation (*R* = 0.68–0.74). ACSH and ASGH showed high similarity based on the chemical genetic interaction score (*R* = 0.89 for AC-produced hydrolysate, and *R* = 0.8 for NAC-produced hydrolysates) (Fig. 2c, d), indicating that most yeast mutants grow similarly in these two different hydrolysates.

Overall, the biological response to the different AC vs. NAC methods was consistent. The deletion mutant of the ATP-binding cassette (ABC) transporter *PDR12* was the most sensitive mutant across all tested hydrolysates (Fig. 2e, v; Additional file 2: Table S2). This gene is a weak acid (e.g., benzoate) inducible multidrug transporter/efflux pump required for weak organic acid resistance [42]. Deletion of *PDR12* causes sorbate and acetate sensitivity [43]. The comparatively high concentrations of benzoic acid in all hydrolysates may also be one reason for the high sensitivity of the *PDR12* knockout mutant [42]. Benzoic acid is known to permeate the yeast cell membrane and requires active transport to be removed from the cell, and under glucose-depleted conditions, significant amounts of benzoic acid can accumulate within the cell [44]. The mutant *snf6*∆ was also consistently sensitive to all hydrolysates (Fig. 2e, vi). *SNF6* is a subunit of the SWI/SNF chromatin remodeling complex, whose deletion confers sensitivity to oxidative stress, as well as other inhibitors such as acetate and ethanol [45–47]. *DPH2* is a poorly characterized gene involved in diphthamide biosynthesis, a post-translational modification of elongation factor 2 (encoded by *EFT1* or *EFT2*) that localizes to the ribosome [48]. *MRPS9* is a subunit of the mitochondrial ribosome, suggesting a need for basic mitochondrial function in hydrolysates even under strict anaerobic conditions [49].

Interestingly, we also noticed several feedstock-specific gene responses that may shed light on inherent chemical differences in the plant biomass. The knockout mutant for *NPT1*, a nicotinate phosphoribosyltransferase involved in the salvage pathway of NAD+ biosynthesis, was uniquely sensitive to ASGH (Fig. 2e, i). Also, knockout mutants of *SIP3* and *ERG3* were specifically sensitive to ASGH, compared to ACSH (Fig. 2e, ii and iv; Additional file 2: Table S2). *SIP3* is a putative sterol transfer protein thought to be involved in retrograde transport of sterols from the plasma membrane to the endoplasmic reticulum. *ERG3* is a C-5 sterol desaturase involved in the ergosterol biosynthesis [50]. Further, in non-autoclaved ASGH, we detected significant enrichment among the top 20 most sensitive mutants for genes involved in the sterol biosynthetic process (*p* < 0.01: *ERG2*, *SLC1*, *TOR1*, *SUR1*, *ERG3*, *ERG6*). These data suggest that ASGH has particular toxicity towards sterol biosynthesis, or is lacking in nutrients required for sterol biosynthesis.

Translation-related deletion mutants were frequently resistant across all hydrolysates (Fig. 2e, iii). In both AC and NAC ASGH we detected enrichment (*p* < 0.05 and *p* < 0.0001, respectively) among resistant mutants related to cytoplasmic translation (e.g., *RPS16B*, *RPL19A*, *RPL22A*, *RPS16A*, *RPL11B*; Additional file 2: Table S2). This enrichment was not detected when mutants were grown in ACSH. Deletion of many of these genes is known to confer increased oxidative stress resistance and longevity [51]. These genes indicate oxidative stress may be a greater factor in ASGH compared to ACSH and may be used to tailor tolerance to ASGH hydrolysates.

Overall, the primary chemical stresses in all hydrolysates seem to be oxidative and weak acid stresses as suggested by chemical genomic signature (e.g., *pdr12*∆ sensitivity in all hydrolysates), and to be very similar in AC and NAC hydrolysates. Sterol biosynthesis may be particularly important for ASGH tolerance, because the chemical genomics showed significant enrichment for ergosterol biosynthesis genes among the top NAC-ASGH sensitive strains (e.g., *erg2∆*, *erg3*∆, *erg6*∆).

### Fermentation of *S. cerevisiae* and *Z. mobilis* in ACSH and ASGH

**Fig. 3:**
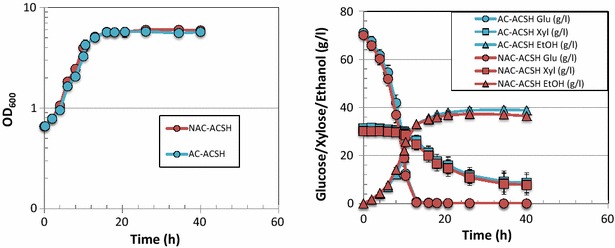
Comparative fermentation of *Z. mobilis* in ACSH produced by AC or NAC methods. *Left panel* cell growth data; *right panel* glucose (*circle*), xylose (*square*), and ethanol (*triangle*) data

**Fig. 4:**
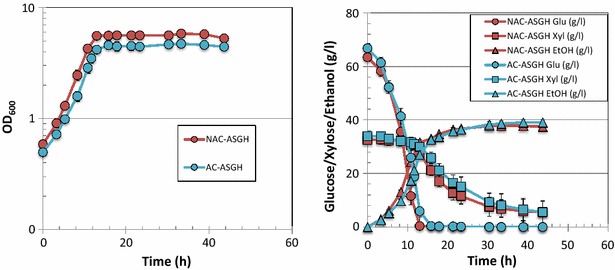
Comparative fermentation of *Z. mobilis* in ASGH produced by AC or NAC methods. *Left panel* cell growth data; *right panel* glucose (*circle*), xylose (*square*), and ethanol (*triangle*) data

In order to understand better the difference of microbial fermentation performance in the different hydrolysates, we used two engineered xylose-utilizing ethanologens, *S. cerevisiae* Y128 and *Z. mobilis* 2302 to evaluate differences in cell growth, glucose and xylose utilization, and ethanol production. As shown in Fig. 3, *Z. mobilis* showed very similar growth in ACSH produced by the two different methods, with OD_600_ reaching ~5 by the time glucose had been completely utilized at ~13 h. As shown in Table 2, cell growth rate, glucose and xylose uptake rates, and ethanol yield were very similar in ACSH produced by either the AC or NAC method. *Z. mobilis* also showed very similar growth, glucose and xylose utilization, and ethanol yield in switchgrass hydrolysates produced by AC and NAC methods (Fig. 4; Table 2), with a slight difference in cell growth and sugar utilization that was likely due to lower initial inoculation in AC-produced ASGH than NAC-ASGH. Comparison of the two different feedstocks showed no significant difference in cell growth, sugar utilization, and ethanol production when *Z. mobilis* was grown in ACSH and ASGH (Additional file 3: Figure S2; Table 2).

**Table 2:** Growth, glucose and xylose utilization, and ethanol yield by *Z. mobilis* when grown in ACSH and ASGH produced by autoclaved (AC) or no-autoclaved (NAC) methods

	Hydrolysates			
	AC-ACSH	NAC-ACSH	AC-ASGH	NAC-ASGH
Exponential growth rate^a^	0.19 ± 0.01	0.18 ± 0.01	0.19 ± 0.01	0.19 ± 0.01
Exponential glucose uptake rate^a^	12.6 ± 0.8	12 ± 1	13.0 ± 0.5	12 ± 1
Stationary xylose uptake rate^b^	1.1 ± 0.1	1.2 ± 0.1	1.4 ± 0.2	1.1 ± 0.1
Total xylose consumed (mM)^c^	158 ± 12	146 ± 8	172 ± 2	166 ± 4
Total ethanol produced (mM)^c^	850 ± 23	810 ± 22	833 ± 21	819 ± 23
Ethanol yield (%)^d^	82 ± 1	81 ± 1	81 ± 1	83 ± 2

Each value is from at least three biological replicates in different bioreactors

^a^Exponential phase is between 4 and 13 h. Growth rate is per hour, and unit for glucose uptake rate is mM/OD_600_/h

^b^Stationary phase when glucose is gone is between 16 and 30 h. Unit for xylose uptake rate is mM/OD_600_/h

^c^Total xylose consumed and ethanol yield is calculated between 0 and 30 h. All glucose was used at this time point

^d^Calculated from the total ethanol produced and the total glucose and xylose consumed, assuming 2 ethanol per glucose and 1.67 ethanol per xylose

**Fig. 5:**
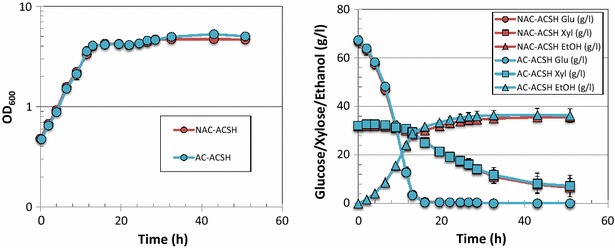
Comparative fermentation of *S. cerevisiae* in ACSH produced by AC or NAC methods. *Left panel* cell growth data; *right panel* glucose (*circle*), xylose (*square*), and ethanol (*triangle*) data

**Fig. 6:**
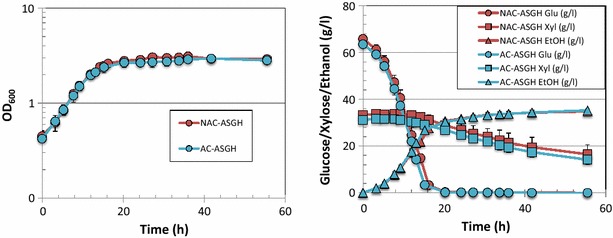
Comparative fermentation of *S. cerevisiae* in ASGH produced by AC or NAC methods. *Left panel* cell growth data; *right panel* glucose (*circle*), xylose (*square*), and ethanol (*triangle*) data

Like *Z. mobilis*, *S. cerevisiae* showed rapid growth and glucose utilization in ACSH, with OD_600_ reaching ~4 when glucose was completely utilized at ~16 h (Fig. 5). There were also no significant differences in the cell growth rate, sugar utilization rate, and ethanol yield in the ACSH batches produced by the two different methods (Table 3). *S. cerevisiae* also showed similar growth, glucose and xylose utilization, and ethanol production in ASGH produced by the two different methods (Fig. 6; Table 3). However, unlike results seen with *Z. mobilis*, *S. cerevisiae* showed significantly slower growth and xylose utilization in ASGH than in ACSH (Table 3; Additional file 4: Figure S3). The growth rate in ASGH was only about 60 % of that seen in ACSH. Because of a lower cell density in ASGH, the glucose utilization rate was slightly higher in ASGH than ACSH. The ethanol yield was also slightly higher in ASGH than ACSH, most likely due to the lower cell growth in switchgrass hydrolysate, so that more energy is funneled into ethanol production rather than into cell mass (Table 3).

**Table 3:** Growth, glucose and xylose utilization, and ethanol yield by *S. cerevisiae* when grown in ACSH and ASGH produced by autoclaved (AC) or no-autoclaved (NAC) methods

	Hydrolysates			
	AC-ACSH	NAC-ACSH	AC-ASGH	NAC-ASGH
Exponential growth rate^a^	0.17 ± 0.01	0.16 ± 0.01	0.10 ± 0.01	0.10 ± 0.01
Exponential glucose uptake rate^a^	13.8 ± 0.7	13.5 ± 0.2	17 ± 2	16 ± 1
Stationary xylose uptake rate^b^	1.2 ± 0.3	1.4 ± 0.2	1.1 ± 0.1	1.0 ± 0.2
Total xylose consumed (mM)^c^	145 ± 7	140 ± 17	90 ± 14	92 ± 21
Total ethanol produced (mM)^c^	790 ± 30	760 ± 20	746 ± 6	722 ± 23
Ethanol yield (%)^d^	80 ± 1	78 ± 1	87 ± 3	85 ± 1

Each value is from at least three biological replicates in different bioreactors

^a^Exponential phase is between 4 and 13 h for ACSH and between 5 and 15 h for ASGH. Growth rate is per hour, and unit for glucose uptake rate is mM/OD_600_/h

^b^Stationary phase when glucose is gone is between 16 and 30 h for ACSH and between 20 and 42 h for ASGH. Unit for xylose uptake rate is mM/OD_600_/h

^c^Total glucose and xylose consumed and ethanol yield is calculated between 0 and 30 h for ACSH and between 0 and 42 h for ASGH. All glucose was used at this time point

^d^Calculated from the total ethanol produced and the total glucose and xylose consumed, assuming 2 ethanol per glucose and 1.67 ethanol per xylose

For all hydrolysates the growth of *Z. mobilis* and *S. cerevisiae* nearly reached a plateau upon exhaustion of the glucose, and the OD_600_ only increased slightly during xylose utilization. Xylose utilization also occurred only when glucose was completely exhausted, indicating that xylose utilization is hindered by the presence of glucose, which could occur through a block in transport or other regulatory mechanisms. Consistent with the former, in *Z. mobilis,* glucose facilitated diffusion protein (Glf) is a shared transporter for both xylose and glucose, but has lower affinity for xylose [52]. A similar transporter exists in *S. cerevisiae* [53]. During co-fermentation of mixed sugars, low levels of xylose utilization could be attributed either to competitive interference with glucose for the transporters or slower metabolism of xylose within the organism [52, 53]. In addition to the delay, there was also incomplete xylose utilization with only 2/3 of the sugar consumed after 48 h of fermentation (Fig. 3). The reason for the decreased xylose utilization rate in late stationary phase (after ~40 h) is unknown; it could be due to cell aging, synergistic effects of end products (such as ethanol) with other inhibitors in the media, insufficiency of ATP synthesis to maintain cell activity, or redox imbalance during xylose metabolism. Further investigation including gene expression and metabolite analysis will be needed to elucidate the bottleneck of xylose utilization in these hydrolysates.

Previously, we studied the growth and sugar utilization of an *E. coli* ethanologen in ACSH [12, 54]. *E. coli* grew robustly in ACSH during the early portion of fermentation. However, cell growth arrested prematurely, despite the presence of abundant glucose. Growth-arrested cells still remained metabolically active and continued to use glucose to produce ethanol. Growth arrest is probably due to the depletion of amino acids or other growth factors [54]. In contrast, both *Z. mobilis* and *S. cerevisiae* showed robust growth in ACSH until the glucose was depleted with OD_600_ reaching ~5 within 13 h, and ~4 within 16 h, respectively (Figs. 3, 5). This indicates that there are no growth limiting factors for *Z. mobilis* and *S. cerevisiae* in these hydrolysates during the glucose consumption phase and growth was mainly dependent on the glucose concentration.

*Saccharomyces cerevisiae* showed significantly slower growth and xylose utilization in ASGH than in ACSH, while no significant difference was seen in *Z. mobilis*. Interestingly, most inhibitors, including lignotoxins, tend to be lower in ASGH than ACSH. However, both benzoic acid and benzamide are present at significantly higher levels in ASGH than ACSH. Benzoic acid is known to permeate yeast cell membranes and requires active transport to be removed from the cell. When yeast are grown under glucose-depleted conditions, significant amounts of benzoic acid can accumulate [44]. The required redirection of ATP toward active export could reduce cell growth [55–57]. The comparatively high concentrations of benzoic acid in all hydrolysates may also be one reason for the high sensitivity of the *PDR12* knockout mutant, which has lost a weak acid (i.e., benzoate) inducible transporter and may experience high levels of benzoate accumulation [57]. This accumulation following glucose depletion could be one reason for the slow cell growth of *S. cerevisiae* in ASGH during the xylose utilization phase. However, the slow cell growth and poor xylose utilization could also be due to lower levels of some important required components that were initially missing or had become depleted during the glucose consumption phase, including sterol compounds or amino acids, rather than due to a high concentration of one or more inhibitors.

Overall, primary chemical stresses in all hydrolysates seem to be oxidative and weak acid stresses as suggested by the chemical genomics, but to be similar in AC vs NAC hydrolysates. Sterol biosynthesis may be particularly important for ASGH tolerance, since the chemical genomics showed significant enrichment for sterol biosynthesis genes among the top NAC-ASGH sensitive strains. New inhibitory compounds are still being discovered from hydrolysates, and it may be that an uncharacterized or unquantified compound unique to ASGH could explain the differences. Understanding this effect will require expanding the chemical analysis of hydrolysates and then conducting fermentation experiments with supplementation of growth factors or addition of inhibitors. Future experiments will also be needed to investigate the feedstock-specific chemical landscape found in hydrolysates.

## Conclusion

In this work, we compared hydrolysates produced from two different feedstocks using two different methods. As summarized in Table 4, both the AC and NAC methods have their advantages and disadvantages. The use of antibiotics in large-scale fermentations also carries potential financial and environmental costs. Furthermore, although most antibiotics have no effect on yeast, many bacteria, including *Z. mobilis*, lack resistance to antibiotics thus limiting their use in hydrolysate production or fermentations where they could inhibit the fermentation microbe. We used gentamicin, to which *Z. mobilis* is naturally resistant, during hydrolysis in this study. Gentamicin was able to control contamination in both year-2012 ACSH and year-2010 ASGH effectively. However, we found that the gentamicin did not consistently limit contamination in ACSH produced from corn stover harvested in different years, and was particularly ineffective with corn stover harvested in 2010 (Fig. 1), for which visual analysis of the untreated feedstock and ash analysis indicated higher than usual soil contamination. Although the AC method for hydrolysate production would likely not be used for large, industrial-scale cellulosic ethanol production due to higher capital and operating costs, it is extremely useful for generating hydrolysate of consistent quality as a reagent for laboratory experiments. This is due both to its ease of implementation, as well as the ability to reliably eliminate contamination without the use of antibiotics, while maintaining consistent concentrations of sugars in a variety of hydrolysates. Although autoclaving AFEX-treated biomass prior to hydrolysis increased the concentration of some lignocellulose-derived inhibitors, it had no significant effect on the fermentation performance of either *Z. mobilis* or *S. cerevisiae*. Similar growth, sugar utilization, and ethanol production was seen in hydrolysates produced using both the autoclaved and non-autoclaved methods.

**Table 4:** Advantage and disadvantage of AC vs. NAC methods

Methods	Autoclaved biomass (AC method)	Non-autoclaved biomass, with antibiotics (NAC method)
Operation time	Same	Same
Control of contamination	Completely	Variable (depending on the feedstocks)
Quality of hydrolysate (glucose and lactate)	Very similar	Variable
Concentration of inhibitors	Most are higher than non-autoclaved ones	Most are lower than autoclaved ones
Useful for yeast studies	Yes	Yes
Useful for *Zymomonas* studies	Yes	Variable (depending on strains and antibiotics used)
Industrial SOP	No	Probable
Environmental issues	None	Development of antibiotics resistance

## Methods

### Feedstock production

The feedstocks were cultivated at the Arlington Agricultural Research Station (ARL, 43°17′45″N, 89°22′48″W, 315 masl). Corn stover was sourced from Arlington field 570 (ARL-570) and switchgrass from ARL-346. The main soil at ARL is Plano silt-loam (fine-silty, mixed, superactive, mesic Typic Argiudoll); a deep (>1 m), well-drained mollisol developed over glacial till and formed under tallgrass prairie [58]. Mean annual temperature and precipitation are 6.9 °C and 869 mm, respectively [59, 60].

Pioneer 36H56 corn stover (triple stacked with Roundup Ready and corn borer and rootworm resistance) was planted on May 11, 2012. Starter fertilizer (18-46-0 Diammonium Phosphate) was applied on 10 May and 28 % Urea Ammonium Nitrate was applied on June 7, 2012. A mixed pre-emerge herbicide (2,4-D LV4 Ester; Glyphosate; Mesotrione; S-Metolachlor: 21 oz/acre) was applied on April 16, 2012 prior to planting and a mixed post-emerge herbicide (Glyphosate; Tembotrione; Ammonium Sulfate; Methylated Seed Oil: 24 oz/acre) on June 8, 2012. Corn stover was collected shortly after grain harvest in early October 2012 using a combine that had been modified to separate the corn grain and then chop and bail the corn stover. Following harvest, materials were dried in a 60 °C oven until dry weight was stable, milled using a 18-7-301 SchutteBuffalo hammer mill (SchutteBuffalo, Buffalo, NY, USA) equipped with a 5-mm screen, and stored at room temperature in sealed bags until use.

Switchgrass (Shawnee variety) was planted on May 29, 2004 using a Brillion Sure Stand seeder (Landoll Corporation, Marysville, KS, USA) at a rate of 16.8 kg/ha. For initial weed control, Quinclorac herbicide (585 mL Al/ha) was applied 1 day after planting. A tank mix of Imazethapyr (105 mL Al/ha) and Dicamba (585 mL Al/ha) was applied on May 19, 2006 for additional weed control. Each year in April granular urea (46-0-0) was top dressed at a rate of 90 kg/ha [61]. In mid-October 2010, switchgrass was cut and conditioned with a 4.5-m wide hay-bine (John Deere 4990). Following harvest materials were collected and dried in a 60 °C oven until dry weight was stable, milled using a Christy Turner mill (Christy Turner Ltd., Suffolk, UK) equipped with a 2-mm screen, and stored at room temperature in sealed bags until use.

We also used some corn stover and switchgrass harvested from different years to test the effectiveness of control of contamination by antibiotics. These feedstocks were grown in the same location, but harvested in three different years (2010, 2012 and 2013).

### Preparation of AFEX-pretreated corn stover and switchgrass

AFEX pretreatment was adapted from a previously described method [17]. Immediately prior to performing the pretreatment, water (0.67 or 0.60 g H_2_O:g dry biomass, for corn stover or switchgrass, respectively) was added to milled corn stover (750 g dry weight) or switchgrass (700 g dry weight). The biomass was mixed by hand and then loaded in a 5-gallon stainless steel reactor equipped with an anchor stirrer with attached PTFE wiper blades (Model 4557: Parr Instrument Co., Moline, IL, USA) and kept inside a fume hood. Once sealed, the reactor was charged with nitrogen to 60 psi and stirred at ~30 rpm. The reactor was preheated for approximately 30 min for an initial run, to ~41–45 °C or ~48–51 °C for corn stover and switchgrass, respectively. Following the preheating treatment, liquid ammonia (1 or 2 g NH_3_:g dry biomass, for corn stover or switchgrass, respectively) was added to the reactor using a LEWA EK1 metering pump (Leonberg, Germany). Based on preliminary experiments, the ammonia loading was increased for switchgrass to correct in part for its lower digestibility and hydrolysis glucose yields compared to corn stover. The reactor reached the 100 °C set point within 5 min after ammonia loading and was maintained at 100 ± 10 °C of the reaction temperature for the duration of the 30 min residence time. At the end of the reaction, the ammonia was released from the reactor and filtered compressed air was passed over the biomass for approximately 5 min to facilitate removal of residual ammonia. The biomass was then removed from the reactor and dried by passing filtered compressed air over the biomass within a custom fume-vented acrylic drying box, until the biomass moisture content was reduced to ≤12 % (total weight basis). Following drying, the AFEX-treated biomass was packaged into UV-treated bags inside a laminar flow hood and stored at room temperature until used. The compressed air for the initial ammonia evaporation and biomass drying was filtered using sterilized 0.22 μm Opticap^®^ XL4 Durapore^®^ Capsules (EMD Millipore, Darmstadt, Germany).

### Production of corn stover hydrolysate (ACSH) and switchgrass hydrolysate (ASGH)

We previously reported a method for producing 6 % glucan-loading AFEX corn stover hydrolysate (ACSH) from autoclaved pretreated biomass [12, 32]. In summary, for the autoclaved pretreated feedstock method (referred to as the AC method), in order to obtain comparable glucose concentrations, the AFEX-pretreated corn stover or switchgrass were added at 6 % (~19 % solids, adjusted for moisture content), to a 3-L vessel of Applikon *ez*-control bioreactor system (Applikon Biotechnology, Foster City, CA, USA). A predetermined amount of water (roughly 4.6 L, depending on the moisture content of the pretreated feedstock) was then added and mixed in and the entire vessel was autoclaved for 2 h at 121 °C. After cooling to ~50 °C, 5.6 mL of undiluted HCl was added to decrease the initial pH and optimize enzyme activity, and mixed into the slurry by hand shaking the vessel. CTec2 and HTec2 from Novozymes (Franklinton, NC, USA) were added at 32 mg protein/g glucan and 9 mg protein/g glucan. A similar procedure was used for producing AFEX switchgrass hydrolysate (ASGH). However, because of less efficient glucose conversion of switchgrass, the glucan loading was increased to 7 % and the enzyme loading was increased by 1.5-fold compared to corn stover in order to obtain a comparable glucose concentration following hydrolysis. 6 mL of undiluted HCl was added to adjust initial pH. Following addition of the enzymes, the vessel was shaken by hand to mix the enzymes into the slurry. The hydrolysis was carried out at 50 °C for 5 days with an initial stirring speed of 1000 rpm (first 16 h), and the stirring speed was reduced to 700 rpm after partial hydrolysis. The final pH was between 5.0 and 5.5. After the solids were removed by centrifugation at 8200*g* and 4 °C for 10–12 h, the supernatant was filter-sterilized sequentially through 0.5 µm GVS Maine Glass Prefilters (Thermo Fisher Scientific Inc. Waltham, MA, USA) and 0.2 µm 1 L Filter Units (Nalge Nunc International Corporation, Rochester, NY, USA), and stored at 4 °C.

To evaluate antibiotics as the microbial control agent during hydrolysate production, the pretreated feedstock was used as is without autoclaving (referred to as the NAC method). The same 3-L Applikon vessel was autoclaved with water for 2 h, and then cooled to ~70 °C. The same quantity of HCl as used in AC method was added and mixed well with the water, and then the AFEX-pretreated corn stover or switchgrass were added at 6 or 7 % glucan-loading, respectively. When the vessel cooled to ~50 °C, the CTec2 and HTec2 enzymes were added at the same concentration as in the AC method, followed by the addition of the antibiotic gentamicin to a final concentration of 25 μg/mL. The remaining steps of the hydrolysis were identical as stated previously for the AC method.

### Chemical analysis of ACSH and ASGH

The main components in ACSH and ASGH, including glucose, xylose, some organic acids and alcohols, were quantitated using HPLC-RID with an Agilent 1260 Infinity system (Agilent Technologies, Palo Alto, CA, USA) as described previously [12]. Samples were diluted tenfold with ddH_2_O, and injected and eluted isocratically with 0.02 N H_2_SO_4_ at a flow rate of 0.5 mL/min (RID flow cell, 50 °C; column, 50 °C). Analyte concentrations were calculated using Agilent ChemStation software version B.04.03 with reference compounds used to generate a standard curve.

The heavy metals, minerals and anions were quantitated using a Thermo Jarrell Ash IRIS Advantage Inductively Coupled Plasma Optical Emission Spectrometer by the University of Wisconsin Soil Testing Laboratories [62].

The major lignocellulose degradation products (referred as lignotoxins) in ACSH and ASGH were determined by HPLC–MS/MS adapted from a methodology reported previously [63]. Samples of ACSH or ASGH were diluted 10- to 200-fold with water and analyzed directly with an Agilent 1200 series quaternary gradient pump with vacuum degasser and thermostated autosampler coupled to an Agilent 6460A triple quadrupole mass spectrometer operated in a dynamic multi-reaction monitoring mode (d-MRM). The analytical column was an Ascentis Express C18 from Supelco/Sigma-Aldrich (St. Louis, MO, USA), 15 cm × 2.1 μm, 2.7 µm particle size. Mobile phases were (A) 0.1 % formic acid and (B) acetonitrile delivered at a rate of 400 µL/min starting at 2 % B for 5 min then increased to 25 % B over 20 min then to 90 % B in 2 min and maintained for 5 min before being returned to 2 % B for 8 min equilibration prior to the next injection. ESI source conditions were: Gas Temp 300 °C, Gas Flow 11 mL/min, Nebulizer 45 psi, Sheath Gas Temperature 360 °C, Sheath Gas Flow 11 mL/min, Capillary-2000 V, Nozzle-1000 V. Compound-dependent MRM transitions, fragmentor voltage and collision energy were determined from automated flow injections of approximately 1 mM individual reference standards using Mass Hunter Optimizer software. Each compound’s retention time was initially determined by the an injection of a standard solution and entered into the dynamic MRM acquisition method with a time window of 2 min. Individual reference standard solutions were made in 50 % methanol at approximately 1–5 mM and appropriate aliquots of each were combined and diluted to a final volume of 10 mL with water to give a reference standard mixture containing each compound at 100 µM. Aliquots of the mixture were kept frozen at −80 °C until used on the day of analysis to produce twelve 1:2 serial dilutions that were injected into the analytical system, at least five levels bracketing the concentrations found in the samples were used to construct an external standard curve from which concentrations found in the samples were calculated.

For quantitation of furfural and acetamide, head space solid phase microextraction—gas chromatography/mass spectrometry (HS-SPME-GC/MS) was used. A Pegasus 4D GCxGC-TOF MS (Leco Corp. Saint Joseph, MI, USA) with an Agilent 6890A gas chromatograph coupled to the ToF mass analyzer via a heated capillary transfer line, and a Gerstel-LEAP Combi PAL autosampler and sample preparation system with Twister heated sample agitator were used for the automated SPME sampling process and analysis. The autosampler was fitted with an SPME needle holder containing a gray hub divinylbenzene/carboxen/polydimethylsiloxane SPME fiber from Supelco/Sigma-Aldrich. Samples were briefly vortex mixed prior to measuring 250 μL of sample, 250 μL of water (a dilution of ½ relative to the standards), and 500 μL of stable isotope labeled internal standard mixture, and approximately 300 mg of NaCl into a 10-mL screw top headspace vial. Vials were quickly capped with magnetic screw caps with 4-mm PTFE-backed silicone rubber septum for SPME. ChromaTOF software V. 4.50.8.0 from Leco was used for system control during acquisition and for data processing, calibration and calculation of final concentrations. Stable isotope labeled internal standards (SILIS) were added to unlabeled reference standards and analyzed to produce a standard curve of relative concentrations vs. peak areas of the unlabeled calibrants relative to the peak areas of the corresponding SILIS. The extracted ion chromatograms from which the peak areas were obtained allowed measurement of each compound independently from its nearly coeluting SILIS.

Reagents and reference compounds were from Sigma Aldrich Co. (Saint Louis, MO, USA) or Thermo Fisher Scientific Inc. Reference standards of eight ferulic acid dehydrodimers (“Diferulates”) were donated by Dr. John Ralph, University of Wisconsin, Madison, USA. D5-Acetamide, D3-Furfural, D5-furfuryl alcohol for HS-SPME-GC/IDMS were obtained from C/D/N isotopes, Inc. (Pointe-Claire, Quebec, Canada). Vanillamide (3-methoxy,4-hydroxybenzamide) was prepared by treatment of 3-methoxy-4-hydroxybenzonitrile with sodium perborate as previously reported [64]. Coumaroyl amide and feruloyl amide were synthesized as described previously [12].

### Strains, media, growth and fermentation conditions

Engineered xylose-utilizing *S. cerevisiae* Y128 [65] and *Z. mobilis* 2032 (obtained from the American Type Culture Collection, PTA-6977) were used for comparative fermentation. The yeast Y128 was inoculated into 10 mL YPD medium containing 10 g/L yeast extract, 20 g/L peptone, and 20 g/L glucose, with 200 g/L geneticin G418, and grown under aerobic condition at 30 °C. After ~7 h, the cultures were diluted into 60 mL YPD medium containing G418 at an initial OD_600_ of 0.2, and then grown under aerobic conditions at 30 °C overnight. The cultures were diluted into 500 mL of hydrolysate with an initial OD_600_ of 0.5, and then grown in an anaerobic chamber (Coy Laboratory Products, Grass Lake, MI, USA) for about 14 h on a stir plate at 30 °C, and then used to inoculate the fermenters as described later.

Similarly, *Z. mobilis* 2032 was inoculated into 5 mL ZRMG medium (or RM, rich medium) containing 20 g/L glucose; 10 g/L yeast extract; and 2 g/L KH_2_PO_4_ [66], with 12.5 g/L each of tetracycline (Tc) and chloramphenicol (Cm), and grown under aerobic conditions at 30 °C. After ~7 h, the cultures were diluted into 80 mL of RMGX medium containing 100 g/L glucose; 20 g/L xylose; 10 g/L yeast extract; and 2 g/L KH_2_PO_4_, plus 12.5 g/mL each of Tc and Cm to an initial OD_600_ of 0.4, and then grown under aerobic conditions at 30 °C overnight. The cultures were diluted into 500 mL of hydrolysate at an initial OD_600_ of 0.5, and then grown in an anaerobic chamber on a stir plate at 30 °C for about 14 h, and then used to inoculate the fermenters as described later.

Fermentations were conducted in 0.5-L bioreactors (BIOSTAT Q_plus_ system from Sartorius, Bohemia, NY, USA) containing 300 mL of hydrolysate. Prior to fermentation, the hydrolysates were adjusted to pH 5 (*S. cerevisiae*) or 5.8 (*Z. mobilis*) using 10 N NaOH or undiluted HCl and filtered through a 0.2 μ filter to remove precipitates and to ensure sterility. After transfer to the fermentation vessel, hydrolysates were sparged with 100 % N_2_ at the flow rate of 150 mL/min for ~6 h. Fermentations were conducted at 30 °C with continuous stirring (300 rpm) and sparging (150 mL/min; 100 % N_2_). After OD_600_ measurements of the starter cultures, cells were centrifuged at 14,000*g* for 3 min. The supernatant was discarded and the cell pellets were resuspended into 10 mL of hydrolysate from the pre-sparged vessels, and then inoculated back into each vessel. The initial OD_600_ of cells in the bioreactors was 0.5. During the fermentation, pH was controlled at 5.0 for Y128 and 5.8 for *Z. mobilis* 2032 by automated addition of 5 % NaOH.

Cell growth was monitored by removing samples from the bioreactors and measuring OD_600_ using a Beckman Coulter DU720 spectrophotometer (1.3 mm slit width) (Beckman Coulter, Inc., Brea, CA, USA) in a 1-mL cuvette. Depending on the OD_600_, cells were diluted 1:10 or 1:25 with nano-pure water before the measurement, and the background OD_600_ of the fermentation broth was also measured and subtracted to determine the final OD_600_. The concentration of glucose, xylose, and the end products were analyzed using HPLC-RID as described above. To obtain a more accurate quantitation of the ethanol produced, the evaporated ethanol from an off-gas line was trapped in ice water and quantitated using HPLC-RID, to determine the final ethanol yield.

Synthetic hydrolysate media (SynH and SynH2) that mimics 6 % glucan-loading ACSH, has been developed and used in our previous studies [12, 54]. Based on the new chemical analysis data of ACSH, including minerals, amino acids, and many other components, we have modified SynH2 to more closely approximate the composition of ACSH, generating a new SynH2.1. The chemical composition of in SynH2.1 and SynH2 are listed in Additional file 5: Table S1.

### Chemical genomic analysis of ACSH and ASGH

Chemical genomic analysis of these hydrolysates was performed as described previously, using a collection of ~4000 yeast deletion mutants [41, 67]. 200 µL cultures of the pooled collection of *S. cerevisiae* deletion mutants were grown in the different versions of ACSH and ASGH, or synthetic hydrolysate (SynHv2.1) in triplicate for 48 h at 30 °C. Genomic DNA was extracted from the cells and mutant-specific molecular barcodes were amplified using specially designed multiplex primers as described previously [41]. The barcodes were sequenced using an Illumina HiSeq2500 in rapid run mode (Illumina, Inc, San Diego, CA, USA). The barcode counts for each yeast deletion mutant in the hydrolysates were normalized against the synthetic hydrolysate control in order to define sensitivity or resistance of individual strains (chemical genetic interaction score). A resistant mutant has a positive interaction score, whereas a negative score indicates a sensitive mutant. The average chemical genomic interaction scores are provided in the supplemental information (Additional file 2: Table S2). The pattern of genetic interaction scores for all mutant strains represents the chemical genomic profile or “biological fingerprint” of a sample [41, 67]. To correlate chemical genomic profiles, replicates were averaged and correlations calculated using Spotfire 5.5.0 (Tibco, Boston, MA, USA). A Bonferroni-corrected hypergeometric distribution test was used to search for significant enrichment of GO terms among the top 20 sensitive deletion mutants [68]. The clustergram of the chemical genomic profiles was created in Cluster 3.0 [69], and visualized in Treeview (v1.1.6r4) [70].

### Statistical analysis

Statistical analysis of the hydrolysate composition was conducted in R-Studio^®^, version 0.98.1102 (Boston, MA, USA). A linear model of each chemical component was developed based on the feedstock, control method and their interaction, and evaluated using Tukey’s HSD test based on 95 % confidence intervals (Agricolae package, version 1.2-1 [71]). When a reported value was below the limit of quantitation (LOQ), the value was recalculated as LOQ/√2 [72]. These recalculated values were used to determine the mean, standard deviation, and statistical differences.

## Additional files

10.1186/s13068-015-0356-2 Correlation of chemical genomics profiles of ACSH and ASGH produced by AC and NAC methods.
10.1186/s13068-015-0356-2 Average chemical genomic interaction scores for ACSH and ASGH produced by autoclaved (AC) or no-autoclaved (NAC) methods.
10.1186/s13068-015-0356-2 Comparative fermentation of *Z. mobilis* in ACSH and ASGH produced by NAC method. Left Panel: cell growth data; Right Panel: glucose (circle), xylose (square), and ethanol (triangle) data.
10.1186/s13068-015-0356-2 Comparative fermentation of *S. cerevisiae* in ACSH and ASGH produced by NAC method. Left Panel: cell growth data; Right Panel: glucose (circle), xylose (square), and ethanol (triangle) data.
10.1186/s13068-015-0356-2 Composition of SynHv2.1 and SynH2.

## Abbreviations

HMF: 5-hydroxymethylfurfural
YPD: yeast extracts/peptone/glucose medium
ZRMG: *Zymomonas*-rich medium with 2 % glucose
HPLC: high performance liquid chromatography
OD_600_: optical density at 600 nm
CS: corn stover
SG: switchgrass
ACSH: hydrolysate produced using AFEX-pretreated corn stover
ASGH: hydrolysate produced using AFEX-pretreated switchgrass
