# Supplementary material for: Controlling microbial contamination during hydrolysis of AFEX-pretreated corn stover and switchgrass: effects on hydrolysate composition, microbial response and fermentation
Source: Biotechnol Biofuels. 2015 Nov 14;8:180. doi: 10.1186/s13068-015-0356-2 (PMC4650398; doi:10.1186/s13068-015-0356-2)
Supplement: Supplementary file 1 — 10.1186/s13068-015-0356-2 Correlation of chemical genomics profiles of ACSH and ASGH produced by AC and NAC methods. [file 13068_2015_356_MOESM1_ESM.pptx]

## Slide 1
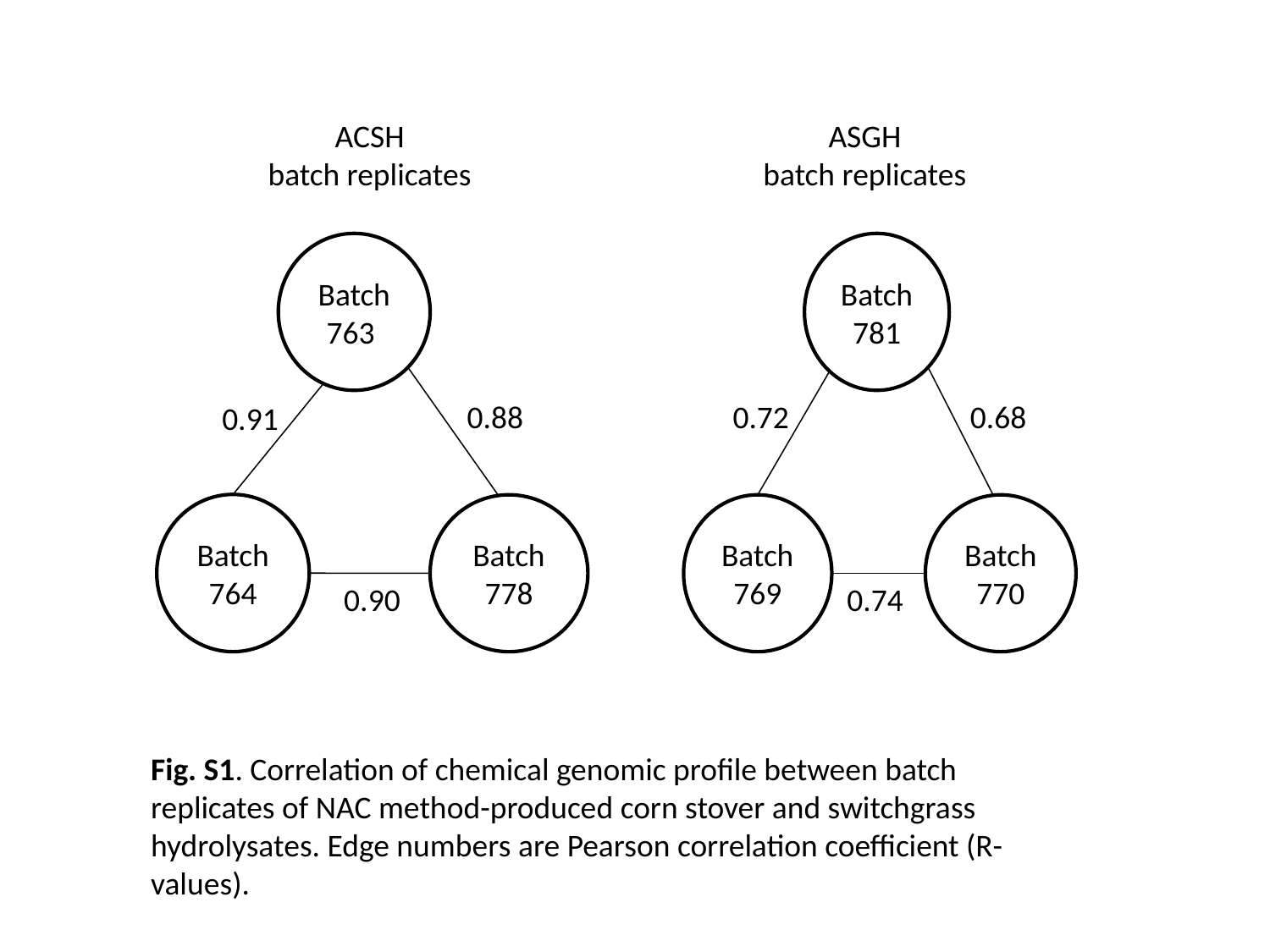

ACSH
batch replicates
ASGH
batch replicates
Batch
763
Batch
781
0.88
0.72
0.68
0.91
Batch 764
Batch 778
Batch 769
Batch 770
0.90
0.74
Fig. S1. Correlation of chemical genomic profile between batch replicates of NAC method-produced corn stover and switchgrass hydrolysates. Edge numbers are Pearson correlation coefficient (R-values).
