# Supplementary material for: Controlling microbial contamination during hydrolysis of AFEX-pretreated corn stover and switchgrass: effects on hydrolysate composition, microbial response and fermentation
Source: Biotechnol Biofuels. 2015 Nov 14;8:180. doi: 10.1186/s13068-015-0356-2 (PMC4650398; doi:10.1186/s13068-015-0356-2)
Supplement: Supplementary file 3 — 10.1186/s13068-015-0356-2 Comparative fermentation of Z. mobilis in ACSH and ASGH produced by NAC method. Left Panel: cell growth data; Right Panel: glucose (circle), xylose (square), and ethanol (triangle) data. [file 13068_2015_356_MOESM3_ESM.pptx]

## Slide 1
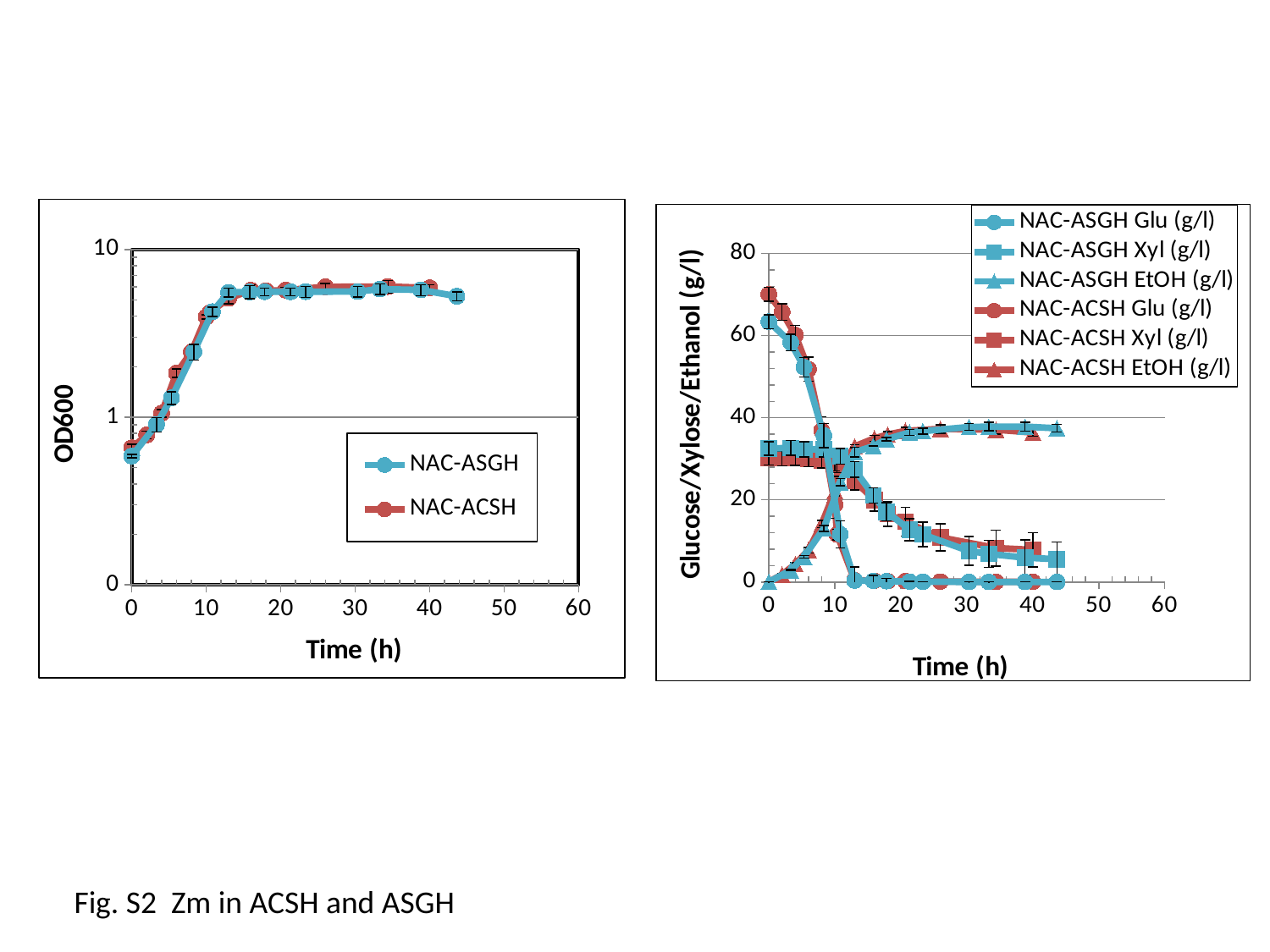

### Chart
| Category | NAC-ACSH | NAC-ASGH |
|---|---|---|
### Chart
| Category | NAC-ACSH Glu (g/l) | NAC-ACSH Xyl (g/l) | NAC-ACSH EtOH (g/l) | NAC-ASGH Glu (g/l) | NAC-ASGH Xyl (g/l) | NAC-ASGH EtOH (g/l) |
|---|---|---|---|---|---|---|Fig. S2 Zm in ACSH and ASGH
