# Supplementary material for: Controlling microbial contamination during hydrolysis of AFEX-pretreated corn stover and switchgrass: effects on hydrolysate composition, microbial response and fermentation
Source: Biotechnol Biofuels. 2015 Nov 14;8:180. doi: 10.1186/s13068-015-0356-2 (PMC4650398; doi:10.1186/s13068-015-0356-2)
Supplement: Supplementary file 5 — 10.1186/s13068-015-0356-2 Composition of SynHv2.1 and SynH2. [file 13068_2015_356_MOESM5_ESM.docx]

## Table S1. Composition of SynHv2.1 and SynH2

| **Media Component** | **SynHv2.1** | | **SynH2** |
| --- | --- | --- | --- |
| **Carbohydrates (mM)** |  |  | |
| D-Glucose | 333 | 333 | |
| D-Xylose | 200 | 200 | |
| L-Arabinose^1^ | 20 | - | |
| D-Arabinose | - | 34 | |
| D-Galactose | 2.9 | 6.4 | |
| D-Mannose | 1.2 | 2.6 | |
| D-Fructose | 24 | 8.2 | |
| **Misc. compounds (mM)** |  |  | |
| Lactate | 4 | 0.5 | |
| Pyruvate | - | 30 | |
| Citrate | - | 10 | |
| Nitrate | 1.1 | 0.1 | |
| Formate | 2.8 | 10 | |
| Malate | - | 10 | |
| Succinate | 0.5 | 0.5 | |
| Acetate | 32 | 33 | |
| Acetamide | 80 | 95 | |
| Glycerol | 4.1 | 3 | |
| Glycine betaine | 0.7 | 0.7 | |
| Choline | 0.3 | 0.7 | |
| Carnitine | 0.3 | 0.2 | |
| **Salts (mM)** |  |  | |
| KH_2_PO_4_ | 5.8 | 3.4 | |
| K_2_HPO_4_ | 11.1 | 6.6 | |
| KCl | 36.8 | 45 | |
| NaCl | 1.3 | 25 | |
| (NH_4_)_2_SO_4_ | 30 | 30 | |
| MgCl_2_ | 12.5 | 1 | |
| CaCl_2_ | 5.5 | 0.09 | |
| **Amino Acids (µM)** |  |  | |
| Alanine | 1172 | 700 | |
| Arginine | 144 | 400 | |
| Asparagine | 228 | 200 | |
| Aspartate | 594 | 350 | |
| Cysteine | 50 | 50 | |
| Glutamine | 259 | 100 | |
| Glutamate | 607 | 450 | |
| Glycine | 378 | 400 | |
| Histidine | 37 | 80 | |
| Isoleucine | 262 | 250 | |
| Leucine | 371 | 360 | |
| Lysine | 175 | 200 | |
| Methionine | 100 | 100 | |
| Phenylalanine | 282 | 200 | |
| Proline | 656 | 225 | |
| Serine | 369 | 275 | |
| Threonine | 310 | 225 | |
| Tryptophan | 50 | 50 | |
| Tyrosine | 424 | 175 | |
| Valine | 202 | 225 | |
| **Nucleobases (µM)** |  |  | |
| Adenine | 50 | 50 | |
| Cytosine | 50 | 50 | |
| Uracil | 50 | 50 | |
| Guanine | 50 | 50 | |
| **Vitamin, Trace elements, and other components (µM)** |  |  | |
| Thiamine-HCl | 0.4 | 10 | |
| Pantothenate | 3 | 10 | |
| p-Aminobenzoic acid | - | 10 | |
| p-Hydroxybenzoic acid | - | 10 | |
| 2,3-di-Hydroxybenzoic acid | - | 10 | |
| CuCl_2_ | 1.9 | 0.01 | |
| CoCl_2_·6H_2_O | 0.03 | 0.03 | |
| H_3_BO_4_ | 23.1 | 10 | |
| (NH_4_)_6_Mo_7_O_2_·4H_2_O | 0.31 | 0.003 | |
| FeCl_3_ | 20 | 17 | |
| ZnCl_2_ | 20 | 12 | |
| MnCl_2_·4H_2_O | 91 | 100 | |
| Pyridoxine | 2.14 | - | |
| Nicotinic Acid | 26.8 | - | |
| Biotin | 0.1 | - | |
| Inositol | 56 | - | |
| Polysorbate 80 (Tween 80) | 1 ml/L | - | |
| Ergosterol | 10 mg/L | - | |

prepare
